# Effect of Electronic Correlations on the Electronic Structure, Magnetic and Optical Properties of the Ternary RCuGe Compounds with R = Tb, Dy, Ho, Er

**DOI:** 10.3390/ma13163536

**Published:** 2020-08-11

**Authors:** Alexey V. Lukoyanov, Lubov N. Gramateeva, Yury V. Knyazev, Yury I. Kuz’min, Sachin Gupta, K. G. Suresh

**Affiliations:** 1 M.N. Miheev Institute of Metal Physics of Ural Branch of Russian Academy of Sciences, 620108 Ekaterinburg, Russia; gramateeva@imp.uran.ru (L.N.G.); knyazev@imp.uran.ru (Y.V.K.); yukuzmin@imp.uran.ru (Y.I.K.); 2 Ural Federal University named after the first President of Russia B.N. Yeltsin, 620002 Ekaterinburg, Russia; 3 Department of Electronic Science and Engineering, Kyoto University, Kyoto 615-8510, Japan; gupta.sachin.2e@kyoto-u.ac.jp; 4 Department of Physics, Indian Institute of Technology Bombay, Mumbai 400076, India; suresh@phy.iitb.ac.in

**Keywords:** intermetallics, electronic structure, magnetic properties, electronic correlations, optical conductivity

## Abstract

In this study, the ab initio and experimental results for RCuGe ternary intermetallics were reported for R = Tb, Dy, Ho, Er. Our theoretical calculations of the electronic structure, employing local spin density approximation accounting for electron–electron correlations in the 4f shell of Tb, Dy, Ho, Er ions were carried in DFT+U method. The optical properties of the RCuGe ternary compounds were studied at a broad range of wavelengths. The spectral and electronic characteristics were obtained. The theoretical electron densities of states were taken to interpret the experimental energy dependencies of the experimental optical conductivity in the interband light–absorption region. From the band calculations, the 4f shell of the rare-earth ions was shown to provide the major contribution to the electronic structure, magnetic and optical properties of the RCuGe intermetallics. The accounting for electron–electron correlations in Tb, Dy, Ho, Er resulted in a good agreement between the calculated and experimental magnetic and optical characteristics.

## 1. Introduction

Ternary intermetallic compounds of the RTX series with R = rare-earth metal, T = d metal, X = p element, were studied intensively during the last years, see recent reviews [1,2,3,4]. A number of interesting magnetic and electric properties of practical and fundamental importance were found in different compounds of the RTX series, including magnetocaloric effect (MCE) [5] of giant [6,7,8,9,10] and large [11,12,13] magnitude and magnetoresistance, an antiferromagnetic to ferromagnetic order transition [14,15] and rich magnetic transition phase diagram [16,17,18]. Very recently, another promising application of some RXT was revealed as intermetallic electrides [19,20]. Most of these properties are related to the coupling of the R-4f electronic states (localized) and other electronic states (itinerant) in the electronic system. In a magnetic transition induced by field, these features are observed accompanied by changes in volume or symmetry of crystal lattice [18,21]. Recently, many new RTX ternary intermetallics have been synthesized and actively investigated for different d metals as T [22,23,24,25,26,27], as well as usual 3d metals in the second position T in RTX [28,29,30,31,32]. The nontrivial behavior of magnetic and transport properties is found experimentally near the magnetic transition temperatures that makes RTX promising for magnetic refrigeration and other applications [33].

The RCuGe series with the rare-earth ions from Tb to Er in hexagonal crystal structure was synthesized and revisited in [34]. Previously, it was found that the RCuGe compounds with the first part of the R row crystallize in the *AlB_2_*-type crystal structure (space group P6/mmm) and with the second part of the R row crystallize in the hexagonal *CaIn_2_*- (space group P6_3_/mmc) or *LiGaGe*-type structure (space group P6_3_mc) [35]. According to neutron diffraction study [36], the magnetic structure of TbCuGe, DyCuGe, HoCuGe, ErCuGe is the sine modulated structure with a wavevector ***k*** = (1/3, 0, 0) with different orientations of moments [36]. The magnetization measurements, heat capacity and electrical resistivity investigations of our intermetallics [34] revealed the presence of an antiferromagnetic (AFM) ordering in the compounds with Néel temperatures T_N_ equal to 11.8 K (TbCuGe), 5.2 K (DyCuGe), 4.7 K (HoCuGe), 4.1 K (ErCuGe) with the high observed effective magnetic moment equal to 10.1 μ_B_ (TbCuGe), 10.8 μ_B_ (DyCuGe), 10.7 μ_B_ (HoCuGe), 9.8 μ_B_ (ErCuGe) [34]. For DyCuGe, HoCuGe, ErCuGe compounds, magnetic, magnetocaloric and magneto-transport measurements revealed the large MCE. The resistivity curves for TbCuGe, DyCuGe, HoCuGe, ErCuGe have metallic behavior with positive magnetoresistance at the lowest temperature. For the higher temperatures, the magnetoresistance was found to change sign and show a non-monotonic field and temperature behavior [34].

In this study, we report theoretical ab initio and experimental studies of spectral characteristics of the RCuGe series for R as Tb, Dy, Ho, Er, including the electronic structure and optical characteristics, as well as magnetic moments of the ions. The optical characteristics are measured over an interval of wavelengths and interpreted using the results of the electronic structure calculations. We demonstrate that the accounting for electron–electron correlations in the Tb–Er 4f shell in the theoretical calculations in necessary to obtain a good agreement with the experimental magnetic and optical characteristics.

## 2. Computational and Optical Methods

The electronic structure of intermetallics was calculated in a self-consistent approach of the linear muffin-tin orbitals basis LMTO [37] method. The orbital basis of the muffin-tin orbitals was comprised of the 6s, 6p, 5d, 4f states for Tb, Dy, Ho, Er; 4s, 4p, 4d electronic states for Ge; 4s, 4p and 3d states for Cu. The muffin-tin spheres had the radii in TbCuGe: Tb—3.7, Cu—2.6 and Ge—2.8 a.u. The muffin-tin spheres had the radii in DyCuGe: Dy—3.6, Cu—2.6 and Ge—2.8 a.u. The muffin-tin spheres had the radii in HoCuGe: Ho—3.6, Cu—2.8 and Ge—2.7 a.u. The muffin-tin spheres had similar radii in ErCuGe: Er—3.7, Cu—2.9 and Ge—2.8 a.u. The differences between these values are caused by the different lattice parameters of the compounds. To perform integration in reciprocal space, a k-points grid with the total number of k-points 512 = 8 × 8 × 8 was taken. The local spin density approximation with U-correction, i.e., LSDA+U (generally referred to as DFT+U) method [38], was used in the computational package [39] without spin–orbit coupling assuming collinear alignment of magnetic moments. Strong 4f electron–electron correlations for Tb–Er were accounted for by the parameter of Coulomb interaction equal to 5.7 (Tb) eV, 5.8 (Dy) eV, 6.5 (Ho) eV, 7 (Er) eV and parameter of exchange interaction of J = 0.7 eV, which are typical for these R ions [40,41]. In [42], very close values of U increasing from 5 to 7 eV were estimated for Tb–Er metals from previous experimental spectroscopic studies and found very close to the values used in the other theoretical calculations [42].

The optical properties of all compounds were carried out using the technique of spectroscopic ellipsometry with rotating analyzer (Beattie method [43]). The ellipsometry is based on the fact that the state of polarization of light is changed on reflection. The investigations were performed in the wavelength range *λ* between 0.22 and 15 μm which corresponds to photon energies *E* = 0.083–5.64 eV at room temperature. The refractive *n*(*λ*) and absorption *k*(*λ*) indices (so-called optical constants) were found from measurements detecting the ratio of the amplitudes and the difference between the phases of s and p polarized light reflected from a surface. The values of *n* and *k* were used to calculate the real *ε*_1_ = *n*^2^ − *k*^2^ and imaginary *ε*_2_ = 2*nk* parts of the complex permeability, optical conductivity *σ* = *ε*_2_ *ω*/4*π* (here *ω* is the frequency of light) and reflectivity *R* = [(*n* − 1)^2^ + *k*^2^]/[(*n* + 1)^2^ + *k*^2^].

## 3. Crystal Structure

**Figure 1:**
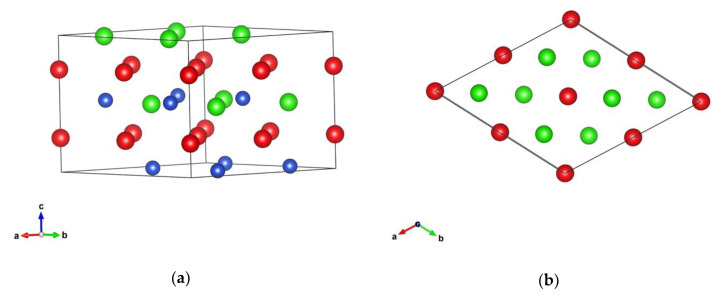
Crystal structure of TbCuGe in a perspective view (**a**) and the projection along the c axis (**b**). Red spheres stand for Tb, green—Ge and blue ones—Cu, which are in (**b**) under the Ge ions.

The RCuGe compounds for R ranging from Tb to Er all have the *LiGaGe*-type hexagonal structure with space group P6_3_mc (#186). The crystal structure of TbCuGe is plotted in Figure 1. For TbCuGe, the parameters of lattice are a = 4.249(9) Å, c = 4.094(9) Å, for DyCuGe—a = 4.231(2) Å, c = 7.179(2) Å, for HoCuGe a = 4.277(9) Å, c = 7.16(9) Å, for ErCuGe—a = 4.44(2) Å, c = 7.08(2) Å [34]. In this structure, there are two formula units within the elementary cell, the R ions are located at 2*a* (0, 0, 1/4) positions, Cu—2*b* (1/3, 2/3, z_Cu_), Ge—2*b* (1/3, 2/3, z_Ge_). In our electronic structure calculations for RCuGe, the experimental parameters were used from work [34].

The arc-melt technique was used to synthesize all the polycrystalline samples. The high purity constituent elements, R (99.9% at. purity), Cu and Ge (99.99% at. purity) were taken in their stoichiometric proportions and melted in the water-cooled copper hearth in presence of high purity argon gas. Samples obtained from arc-melting were kept in the evacuated quartz tubes and annealed for 7 days at the temperature of 750 °C. After 7 days furnace was switched off and samples were allowed to cool. The crystal structure and hence the phase purity of the samples were examined by Rietveld analysis of the X-ray diffraction (XRD, Malvern Panalytical, Malvern, UK) [34] patterns obtained at 300 K. The experimental structural parameters were further taken to the ab initio electronic structure calculations.

## 4. Results

In the theoretical calculations for RCuGe, we assumed the antiferromagnetic alignment of the R magnetic moments in the cell because all experimental results [34] revealed the existence of an AFM ordering in the intermetallics. This solution was found as a stable one during the self-consistent calculations with the large values of the magnetic moments because of the spin polarization of the 4f states of Tb, Dy, Ho and Er. The following values of spin magnetic moments of 5.55 (Tb), 4.86 (Dy), 3.93 (Ho) and 2.97 (Er) μ_B_ were calculated (per R ion), see Table 1.

No magnetic moments were obtained at the Cu and Ge ions. Assuming for R the spin magnetic moment being close to the R^3+^ ones, one can make the following correction for R orbital moment not included in the calculations taking values of orbital moment and Lande factor for R^3+^. Then the total moment is calculated as *J* = *L* − *S*, see in Table 1. For the R^3+^ values of the Lande factor g equal to 3/2, 4/3, 5/4, 6/5, the effective magnetic moment values *g*$\sqrt{J\;\left(J+1\right)}$ are estimated as 9.38, 10.55, 10.56, 9.56 μ_B_ for TbCuGe, DyCuGe, HoCuGe and ErCuGe, respectively, see Table 2. The obtained values of the effective magnetic moment are very close to the pure R^3+^ values of the corresponding R ions and experimental values [34,36].

**Figure 2:**
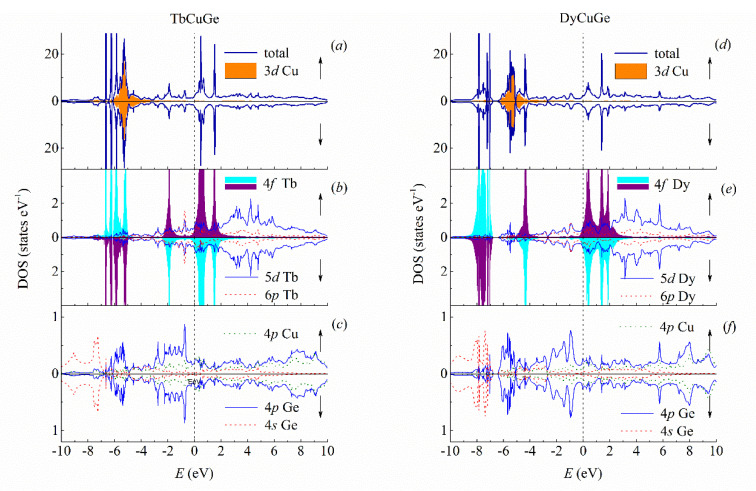
Calculated ab initio densities of states of TbCuGe and DyCuGe. (**a**) Total density of states of TbCuGe and Cu-3d densities; (**b**) densities of states for Tb-4f, Tb-5d and Tb-6p; (**c**) densities of states for Cu-4p, Ge-4p and Ge-4s in TbCuGe; (**d**) total density of states of DyCuGe and Cu-3d densities; (**e**) densities of states for Dy-4f, Dy-5d and Dy-6p; (**f**) densities of states for Cu-4p, Ge-4p and Ge-4s in DyCuGe. Dashed vertical lines show Fermi energy.

The densities of states (DOS) of TbCuGe and DyCuGe calculated for two spin projections are given in Figure 2. One of the main contributions to the valence band in TbCuGe comes from the 3d states of copper located from −6 to −3 eV below the Fermi energy (*E*_F_), see Figure 2a. These electronic states are symmetric in both spin projections due to the zero magnetic moment of Cu in this compound. The narrow peaks are formed by the Tb-4f states near −2 and −7 to −5 eV below *E*_F_ and above *E*_F_ to about 2 eV, see Figure 2b. Because of the antiferromagnetic ordering of the terbium and other R-4f magnetic moments, the corresponding states are strongly spin-polarized and symmetrical for both types of the antiferromagnetically ordered Tb (and other R) ions in the energetic regions corresponding to the occupied and unoccupied orbitals. In TbCuGe, the minor contribution comes from the 5d states of terbium (Figure 2b) with 1.8 e, at the same time, the 4s and 4p states of germanium also have small contributions in this energy interval with 1.2 and 1.8 e, correspondingly, see Figure 2c. All these electronic states are nonmagnetic and do not have spin-polarization. The Fermi energy is located at zero energy.

In DyCuGe, one can distinguish three intervals in the valence band formed mostly by the Dy-4f states at −4.5 and −8 to −7 eV below *E*_F_, as well as a broad band of the Cu-3d states from −6.5 to −3 eV, see Figure 2d. Here the 4f states of the rare-earth ions are located in almost different energy interval from the Cu-3d states. Furthermore, in the valence band, a broad band is formed by the Dy-5d states, see Figure 2e. There are also small contributions to DOS from the 6p states of Dy with 1.3 e, as well as the 4s and 4p states of germanium with 1.2 and 1.8 e and the Cu-4p states with 0.5 e, see Figure 2f.

**Figure 3:**
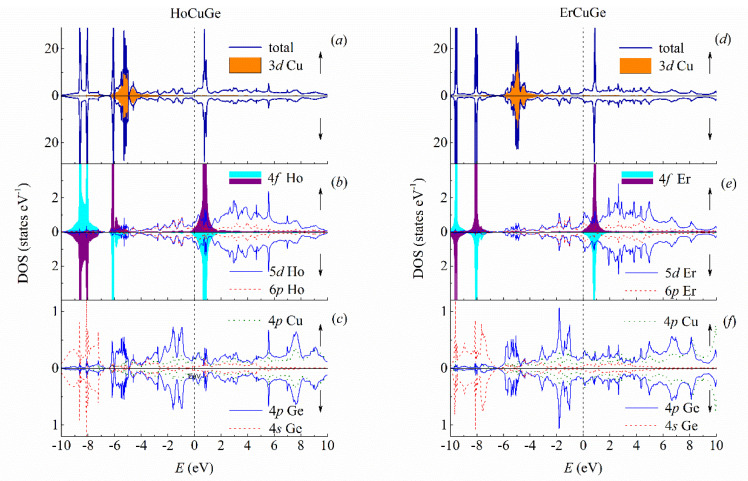
Calculated ab initio densities of states of HoCuGe and ErCuGe. (**a**) Total density of states of HoCuGe and Cu-3d densities; (**b**) densities of states for Ho-4f, Ho-5d and Ho-6p; (**c**) densities of states for Cu-4p, Ge-4p and Ge-4s in HoCuGe; (**d**) total density of states of ErCuGe and Cu-3d densities; (**e**) densities of states for Er-4f, Er-5d and Er-6p; (**f**) densities of states for Cu-4p, Ge-4p and Ge-4s in ErCuGe. Dashed vertical lines show Fermi energy.

In Figure 3, the total and partial DOS are shown for the HoCuGe and ErCuGe intermetallics. The 3d band of copper is filled with electrons and located almost separately from the 4f states of R in the energies from −6 to −4 eV in both compounds, see Figure 3a,d. The valence band is mainly formed by the 4f states of holmium and erbium: at −8.8, −8 and −6 eV in HoCuGe (Figure 3b) and −9.5 and −8 eV below *E*_F_ in ErCuGe (Figure 3e). The conduction band in both intermetallics is formed by the main narrow peak of the 4f states of Ho or Er which can be found near 0.9 eV above *E*_F_ in both intermetallics. In Figure 3b,e, the 4f states of two different R types of ions with antiferromagnetic alignment of the magnetic moments are plotted with different colors. While the 5d states of holmium and erbium mostly contribute to the conduction band, see in Figure 3b,e, the minor contribution to the valence band is formed by the s and p states of copper and germanium, see Figure 3c,f. The number of electrons in these states are close to those in TbCuGe and DyCuGe. The Fermi is located at zero energy. In Figure 3f, one may notice some tiny differences in DOS for two spin projections. In our case, the differences in DOS may be smeared out by additional broadening, but we intentionally keep the sharpness of peaks to show the narrow and sharp peaks of the R-4f states.

**Figure 4:**
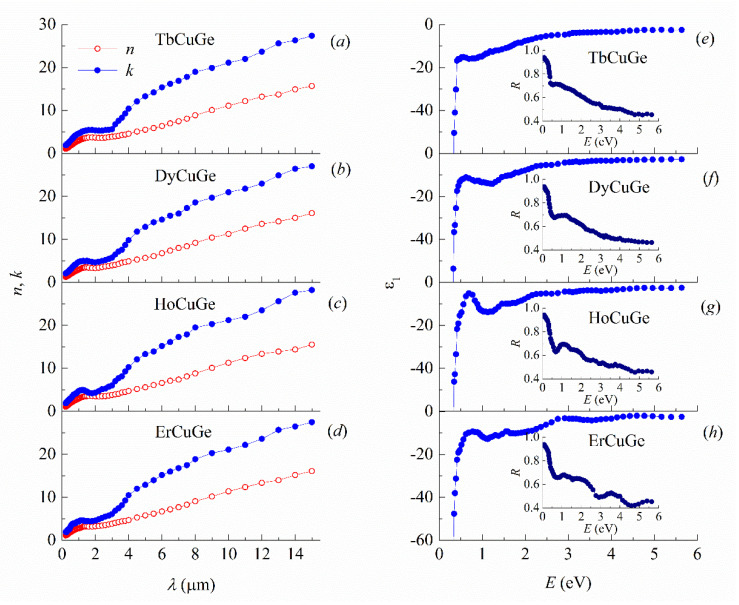
Optical constants *n* and *k* depending on wavelength *λ* for (**a**) TbCuGe, (**b**) DyCuGe, (**c**) HoCuGe and (**d**) ErCuGe. Dependencies of real part of dielectric permeability *ε*_1_ and reflectivity *R* depending on photon energy *E* for (**e**) TbCuGe, (**f**) DyCuGe, (**g**) HoCuGe and (**h**) ErCuGe.

**Figure 5:**
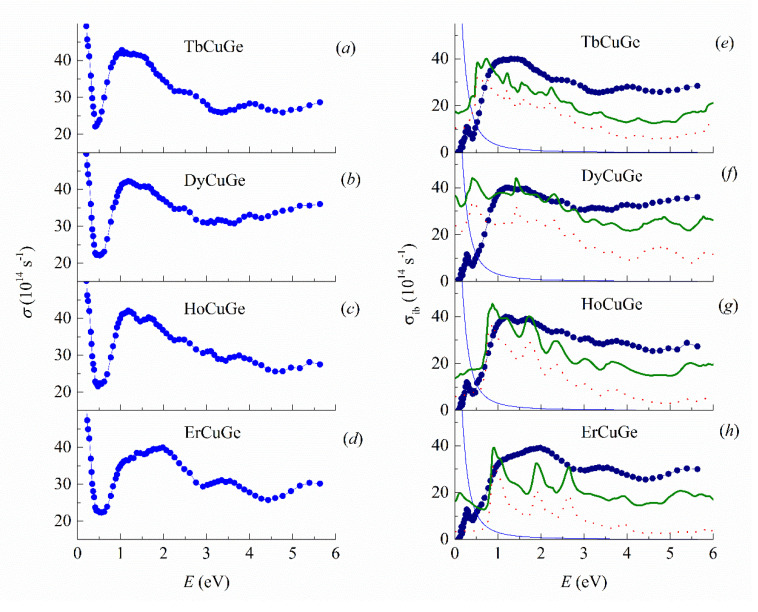
Experimental optical conductivity *σ* depending on photon energy *E* for (**a**) TbCuGe, (**b**) DyCuGe, (**c**) HoCuGe and (**d**) ErCuGe. Experimental (big blue dots) and theoretical (red dots and green curves) interband optical conductivities for RCuGe, with (**e**) R = Tb, (**f**) Dy, (**g**) Ho and (**h**) Er, without the Drude contribution. Solid green curves correspond to the calculated theoretical interband conductivity; contributions of the 5d→4f transitions are shown as red dots; solid light blue lines correspond to estimated Drude part of the optical conductivity for TbCuGe, DyCuGe, HoCuGe and ErCuGe; theoretical curves in (**e**–**h**) are given in arbitrary units.

The optical constants *n* and *k*, real part of dielectric permeability *ε*_1_ and reflectivity *R* for TbCuGe, DyCuGe, HoCuGe and ErCuGe are given in Figure 4. Their energy dependences are inherent to metallic materials. Namely, *k* exceed *n* in the whole interval of wavelengths, and reflectivity is close to unity with the increasing *λ*. In Figure 5, the experimental optical conductivity spectra *σ* are shown for TbCuGe, DyCuGe, HoCuGe and ErCuGe intermetallics, their main peculiarities are rather similar to each other. All measured spectra exhibited typical intraband Drude-like absorption in the long-wavelength region above *λ* > ~2.5 μm (infrared range) and the line shape of *σ*(*ω*) curves resembles the *σ*~*ω*^−2^ dependences. Based on this fact that the free-electron absorption mechanism is dominant in this spectral interval, the plasma frequency *ω*_p_ and effective relaxation frequency of conduction electrons *γ* were obtained from the Drude equations. The relaxation frequency *γ* = *ε*_2_ *ω*/*ε*_1_ characterizes all acts of electron scattering in a metal. In its turn, the plasma frequency *ω*_p_ = (4*πNe*^2^/*m*)^1/2^ defines the value of the collective electron oscillations and ratio of the number *N* of electrons participating in conductivity process, to their effective mass *m*. In the infrared region of 11–15 μm, where contribution of the interband transitions in light absorption is weak, there is no dependence of these parameters on the frequency of light, the values are close to *γ* = 2 × 10^14^ s^−1^ and *ω*_p_ = 5.5 × 10^15^ s^−1^ for the studied compounds.

The shape of *σ*(*ω*) in Figure 5a–d above approximately 0.5 eV demonstrates the dominant role of the interband electron transitions. The optical conductivities spectra for TbCuGe, DyCuGe, HoCuGe and ErCuGe in this energy interval are defined by the very intense and broad bands corresponding to the quantum absorption. Their fine structures are individual for each intermetallic compound. These features reflect the peculiarities of the densities of electronic states on both sides of *E*_F_. For this reason, it is worthwhile to compare the optical conductivities in the interband part obtained as a subtraction of the Drude component *σ*_D_(*ω*) = *ω*_p_^2^*γ*/4*π*(*ω*^2^ + *γ*^2^) from the total experimental spectra *σ*_ib_(*ω*) = *σ*(*ω*) − *σ*_D_(*ω*). Here the corresponding dependencies are calculated based on DOS shown in Figure 2 and Figure 3.

The theoretical curves of *σ*_ib_(*ω*) are shown in arbitrary units for the TbCuGe, DyCuGe, HoCuGe and ErCuGe compounds in Figure 5e–h together with the experimental dependencies. The *σ*_ib_(*ω*) curves were calculated following the general approach [44], estimating the optical absorption as being proportional to the convolutions of DOS both below and above the Fermi energy including the selection rules. The rules require that these DOS belong to the same ion with the same spin projection and orbital quantum numbers differing only by ∆*l* = ± 1. We summed the calculated curves for all ions of the unit cell. The results demonstrate that the main contribution to the interband part of the conductivity for all TbCuGe, DyCuGe, HoCuGe and ErCuGe compounds comes from the R-5d→4f type of transitions. One can also notice that the R-5d and R-4f states convolutions results in the formation of the broad absorption bands whose structures are similar with calculated *σ*_ib_(*ω*). Other types of electron transition, according to calculations, contribute less to the interband absorption. It should be said also that the theoretical optical conductivities were received in accounting of the superposition of the quantum absorption in both spin channels. These dependencies are presented in Figure 5e–h in arbitrary units, because constant matrix elements were assumed during the calculation of them. Generally, comparing calculated and experimental *σ*_ib_(*ω*) curves exhibit the similarities for them in their spectral shape. It was observed that the spectra have an interband absorption region with high intensity and an abrupt edge at *E* < 1 eV and with some of the identical features. On the other hand, there are different intensities and broadening of the maxima of TbCuGe, DyCuGe, HoCuGe and ErCuGe.

## 5. Conclusions

In this work, both ab initio theoretical and experimental studies of the electronic, magnetic and optical properties of RCuGe ternary intermetallics, for R as Tb, Dy, Ho and Er were performed. Our LSDA+U calculations with the U-correction for the 4f states of R resulted in the magnetic moments, densities of states and theoretical optical conductivity. The calculated DOS for each compound were used to calculate the optical conductivities in its interband component as the convolutions of DOS from below and above the Fermi energy. An analysis of DOS demonstrated that predominantly the d and f electronic states are responsible for the structure of the quantum absorption. The energy dependence of the experimental optical conductivities near the electronic transitions is reproduced by the theoretical curves. The main features of the curves are provided by the interband transitions of the 4f-5d electronic states for Tb–Er. Thus, the 4f shell of R substantially contributes to the magnetic and spectral (optical, in particular) properties of the considered intermetallic compounds and the strong electronic correlations in the R-4f states taken into account in our theoretical calculations result in a good agreement between the theoretical and experimental characteristics.

## Figures and Tables

**Table 1:** Calculated spin 2*S*, orbital *L* and total *J* moments, Lande factor *g*.

Compound	2*S*	*L*	*J*	*g*
TbCuGe	5.55	3	5.78	3/2
DyCuGe	4.86	5	7.43	4/3
HoCuGe	3.93	6	7.97	5/4
ErCuGe	2.91	6	7.46	6/5

**Table 2:** Calculated effective magnetic moment μ_eff_ (calc) of rare-earth ions, theoretical magnetic moments μ_eff_ (R^3+^) calculated for R^3+^, experimental values of the effective magnetic moment from [34,36]. All values are in μ_B_.

Compound	μ_eff_ (calc)	μ_eff_ (R^3+^)	μ_eff_ (exp) [34]	μ_eff_ (exp) [36]
TbCuGe	9.38	9.72	10.1	9.80
DyCuGe	10.55	10.63	10.8	10.73
HoCuGe	10.56	10.60	10.7	10.58
ErCuGe	9.53	9.59	9.8	9.75
